# A medium-throughput screen for inhibitors of human metapneumovirus

**DOI:** 10.1177/2040206619830197

**Published:** 2019-02-13

**Authors:** Jennifer C Becker, Sharon J Tollefson, David Weaver, John V Williams

**Affiliations:** 1 Department of Pathology, Microbiology & Immunology, Vanderbilt University School of Medicine, Nashville, TN, USA; 2 Department of Pediatrics, Vanderbilt University School of Medicine, Nashville, TN, USA; 3 Department of Pharmacology, Vanderbilt University School of Medicine, Nashville, TN, USA; 4 Department of Pediatrics, University of Pittsburgh School of Medicine, Children’s Hospital of Pittsburgh of UPMC, Pittsburgh, PA, USA

**Keywords:** Human metapneumovirus, paramyxovirus, high-throughput screen, topoisomerase inhibitors, statins

## Abstract

Human metapneumovirus, a paramyxovirus discovered in 2001, is a major cause of lower respiratory infection in adults and children worldwide. There are no licensed vaccines or drugs for human metapneumovirus. We developed a fluorescent, cell-based medium-throughput screening assay for human metapneumovirus that captures inhibitors of all stages of the viral lifecycle except budding of progeny virus particles from the cell membrane. We optimized and validated the assay and performed a successful medium-throughput screening. A number of hits were identified, several of which were confirmed to inhibit viral replication in secondary assays. This assay offers potential to discover new antivirals for human metapneumovirus and related respiratory viruses. Compounds discovered using the medium-throughput screening may also provide useful probes of viral biology.

## Introduction

Human metapneumovirus (HMPV), a paramyxovirus discovered in 2001, is a major cause of lower respiratory infection in adults and children worldwide.^1–3^ HMPV is closely related to respiratory syncytial virus, which is the leading cause of bronchiolitis and pneumonia in young children.^4^ HMPV causes hospitalization in previously healthy infants and high-risk groups at rates comparable to parainfluenza viruses and influenza virus.^2,5,6^ More than 90% of children become infected before the age of five and HMPV seroprevalence in adults is nearly 100%.^1,7^ There are no licensed vaccines or drugs for HMPV, and thus there is an unmet need for antivirals. We sought to develop a medium-throughput screen (MTS) for inhibitors of HMPV.

## Methods

### Cells, virus, and antibodies

Human bronchial epithelial cells (BEAS-2B, ATCC CRL-9609) were cultured in OptiMEM (Life Technologies) medium containing 2% fetal bovine serum supplemented with amphotericin, gentamicin, and L-glutamine and incubated at 37°C in 5% CO_2_. HMPV isolate TN/94–49 (subgroup A2) was used for all experiments.^8^ Virus was propagated and titrated using LLC-MK2 cells as described previously.^9^ Virus-infected cells (positive signal) were detected by immunofluorescent staining with human monoclonal antibody (mAb) 54G10 against the HMPV fusion (F) protein^10^ and goat anti-human Ig IRDye 800 CW (Li-Cor Biosciences). A potent HMPV-neutralizing human mAb DS7 specific for the HMPV F protein was used as an inhibition control;^10,11^ this mAb was titrated in the screen to exhibit 50% inhibition of virus signal to serve as the mean.

### Compounds

The MTS was performed in duplicate with three compound libraries. The Spectrum Collection (MicroSource Discovery Systems) comprises 2000 compounds with a wide range of biological activities and structural diversity. The NIH Clinical Collection contains a total of 727 compounds previously used in human clinical trials (http://www.nihclinicalcollection.com). Finally, the Bio-active Lipid I Screening Library (Cayman Chemical) contains 846 bioactive lipids. Individual compounds for secondary confirmation of hits were purchased from Sigma, dissolved in DMSO, and diluted in OptiMEM for in vitro testing.

### MTS assay format

BEAS-2B cells were dispensed into black, clear-bottom, 384-well plates (BD Falcon) at a density of 800 cells/well in 20 μL OptiMEM medium using a MultiDrop Combi (Thermo Scientific) and incubated 24 h. Ten microliters of each compound were then added using a Bravo liquid handler (Agilent), resulting in a final drug concentration of 10 µM (0.1% DMSO). Four hours after compound addition, cells were infected with 10 μL of HMPV using the MultiDrop Combi (multiplicity of infection (MOI) of 5 PFU/cell) and incubated for 48 h. Positive and negative controls were included on each plate. After incubation, 70 μL of 10% buffered formalin was added using a MultiDrop Combi, fixed at room temperature for 60 min, and washed 3× with PBS-0.05% Tween (PBS-T). Plates were blocked with 5% nonfat dried milk in PBS-T for 30 min, stained with primary mAb 54G10 for 30 min, washed 3× with PBS-T, stained with secondary anti-human IgG for 30 min, washed 3× with PBS-T, stained with plasma membrane cell dye FM4-64 (Life Technologies) for 10 min, and washed 3× with PBS-T. Plates were read on an Odyssey Infrared Imager (Li-Cor) using the Automatic mode with a dynamic range of 22 bits. Virus signal was read in the 800 nm channel and cell signal in the 700 nm channel. Raw fluorescence data were analyzed using an algorithm to adjust for variability across the controls on each plate and to compensate for the degree of cell toxicity in relation to the degree of viral suppression in each well. We then categorized potential hits as those that exhibited a statistically significant reduction of virus signal ≥2 standard deviations (SD) from the mean (50% inhibition of virus signal by mAb DS7). The entire screen was performed twice in separate runs one week apart.

### Secondary assays

BEAS-2B cell monolayers were treated with compounds and then inoculated with HMPV at an MOI of 5 PFU/cell. Cells were incubated for 48 h in HMPV serum-free growth medium consisting of Opti-MEM with 5 µg/mL trypsin (both from Life Technologies).^12^ Cell viability in secondary assays was performed using the CellTiterGlo assay (Promega) for mevastatin and simvastatin or by staining with FM-64 as in the primary MTS for the other compounds. Monolayers were fixed and viral infectivity read by staining with mAb 54G10^10^ and fluorescent secondary Ab.

## Results

**Figure 1.**
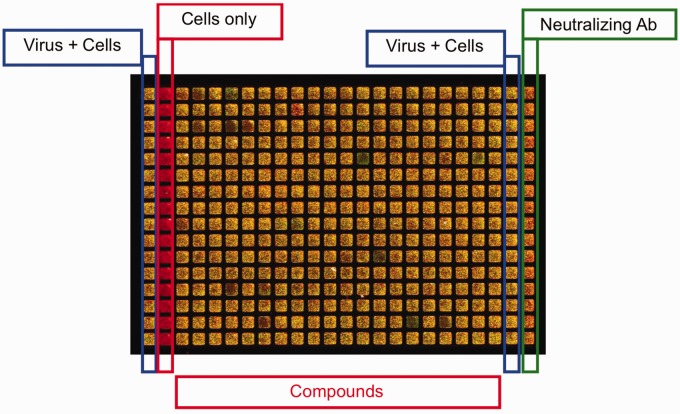
Plate layout for MTS showing image of experimental plate with merged red (cell) and green (virus) fluorescent signals.

Transcription and translation of HMPV proteins occur within 6 h following virus entry, with surface viral protein expression within 12 h and infectious progeny virions produced by 24 h.^12,13^ Therefore, we designed an assay to infect cell monolayers with HMPV and perform readout of infectivity by immunostaining for viral protein expression on the cell surface at 48 h; thus, the assay captured all steps of the virus lifecycle except budding of progeny virions. We included the cell viability dye FM4-64 to normalize signal for cell number and to discriminate specific antiviral effect from cell toxicity. We tested multiple antibody and cell dye concentrations and conditions during optimization (data not shown). The layout of the assay plate is shown in Figure 1.

**Figure 2.**
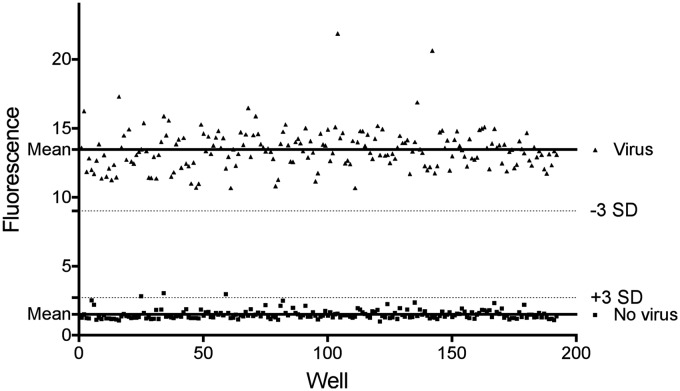
Example plate of raw data used to calculate Z′ for MTS assay. Virus-positive wells are infected with HMPV at MOI 5 PFU/cell; negative wells are uninfected cells.

The sensitivity and dynamic range were determined using serial dilutions of neutralizing mAb DS7 against HMPV as a positive control for inhibition. DS7 exhibited micromolar activity, with 60% neutralization at 1.1 µg/mL and complete inhibition of HMPV at >3 µg/mL, as previously shown.^10,11^ To establish the reproducibility of the assay, we measured fluorescence in cells infected with HMPV and treated with or without neutralizing mAb DS7, performed in triplicate on different days, allowing us to determine well-to-well, plate-to-plate, and day-to-day variation. We used these data to measure the mean fluorescence from mAb-treated and untreated samples for determination of the coefficient of variation, which was ±0.17% for negative signal (cells alone) wells and ±0.01% for positive signal (cells + virus) wells. A key measure of the suitability of a screening assay is the Z′, defined by the formula: Z′ = 1– [(3× (standard deviation of positives) + (3× (standard deviation of negatives)]/(Mean positive–mean negative).^14^ The Z′ of the assay was 0.53, within the desired range of 0.5–1^15^ (Figure 2).

**Figure 3.**
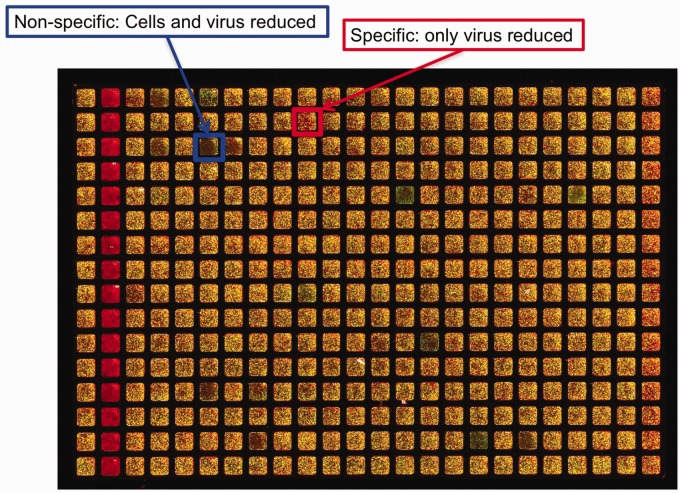
Compound test plate from screen. Layout of plate is identical to Figure 1.

Determination of reduced signal due to cell toxicity was a key aspect of the assay. Some compounds were toxic and reduced virus signal non-specifically as a consequence of reducing cell signal (Figure 3). However, some compounds exhibited a clear virus-specific effect, with a reduction of virus signal but retained cell signal (Figure 3).

Fifty-one compounds were identified that statistically reduced virus signal by ≥2 SD from the mean (Supplemental Table 1). Most of the candidate hits fell into three main categories: (a) 3-hydroxy-3-methyl-glutaryl-coenzyme A (HMG-CoA) reductase inhibitors, or statins; (b) DNA topoisomerase inhibitors; and (c) host factor inhibitors. Several of the compounds identified have been reported to exhibit in vitro activity against other RNA viruses (Table 1), and some were hits in both biological replicates of the screen.

**Table 1.** Selected compounds of interest identified in the MTS for HMPV inhibitors.

Compound	Class	Virus with reported antiviral effect	Ref.
**Simvastatin**	HMG-CoA reductase inhibitor	Influenza	^16–19^
Itavastatin	HMG-CoA reductase inhibitor	Influenza	
**Mevastatin**	HMG-CoA reductase inhibitor	Influenza	
Acacetin	Flavonoid	Influenza	^20^
**Aurintricarboxylic acid**	Topoisomerase II inhibitor	SARS-CoV, HCV, influenza, enterovirus 71	^21–24^
**Irinotecan**	Topoisomerase I inhibitor		
**Etoposide**	Topoisomerase II inhibitor		
**Mitoxantrone**	Topoisomerase II inhibitor		
Teniposide	Topoisomerase II inhibitor		
**Chlorprothixene**	Antipsychotic/endocytosis inhibitor	HSV2	^25^
**Ciclopirox**	Deoxyhypusyl hydroxylase inhibitor	HIV-1	^26^
**Isoproterenol**	Beta-2 receptor agonist	Rotavirus	^27^

Note: Compounds in bold font were hits in both replicates of the MTS.

HMG-CoA: 3-hydroxy-3-methyl-glutaryl-coenzyme A.

**Figure 4.**
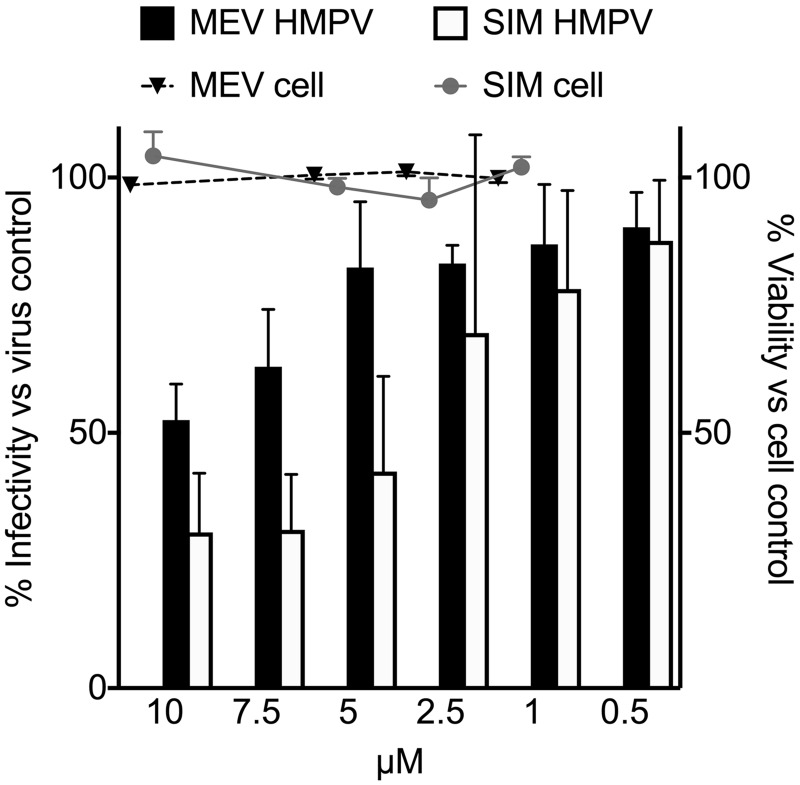
In vitro inhibitory activity of mevastatin (MEV) and simvastatin (SIM). Cell monolayers were treated with the compounds or DMSO control at the indicated concentrations, infected with HMPV, and infectivity scored by fluorescence at 48 h (left y axis, bar indicates mean ± SD). MEV and SIM were tested for cytotoxicity using the CellTiterGlo assay (Promega) and the result plotted on the right y axis (dots, mean ± SD). HMPV: human metapneumovirus.

**Figure 5.**
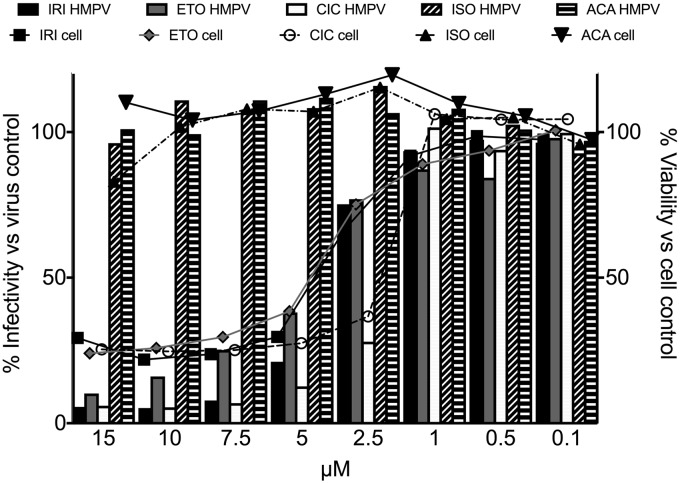
In vitro inhibitory activity of irinotecan (IRI), etoposide (ETO), ciclopirox (CIC), isoproterenol (ISO), and acacetin (ACA). Cell monolayers were treated with the compounds or DMSO control at the indicated concentrations, infected with HMPV, and infectivity scored by fluorescence read at 800 nm at 48 h. Cell viability was measured by cell signal read at 700 nm. HMPV: human metapneumovirus.

To confirm whether the candidates exhibited specific activity against HMPV, we tested serial dilutions of selected compounds from Table 1 in a single-cycle viral growth assay, measuring virus signal and cell viability signal. Compounds were chosen to be tested in secondary assays based on biologic plausibility (HMG-CoA reductase inhibitors), frequency of hits in the compound class (topoisomerase inhibitors), or prior reports of antiviral activity. Mevastatin and simvastatin exhibited a modest, dose-dependent antiviral effect against HMPV but no cellular toxicity in this dosing range by luminescent cell viability assay (Figure 4). Acacetin and isoproterenol exhibited no significant antiviral or cytotoxic effect in the secondary assay (Figure 5). However, irinotecan, etoposide, and ciclopirox exhibited dose-dependent cytotoxicity but modest inhibition of HMPV in addition to the cytotoxicity (Figure 5).

## Discussion

We designed and performed an MTS for inhibitors of HMPV, an important human pathogen. The assay exhibited an acceptable Z′ score and was amenable to automation, showing feasibility to screen much larger compound libraries. We discovered a number of compounds with antiviral activity, several of which demonstrated dose-dependent inhibition of HMPV independent of cellular toxicity.

We were particularly intrigued by the multiple statins identified in the screen. HMG-CoA reductase inhibitors (statins) are associated with milder disease and improved outcomes of influenza infection in humans, although the mechanism is unknown and clinical trials have offered conflicting results.^16–19^ Statins have been shown to inhibit endocytosis of influenza virus^28^ and HMPV enters cells by endocytosis,^29^ making the identification of statins and the endocytosis inhibitor chlorprothixene interesting. Indeed, statins inhibited HMPV infection by ∼50%. However, HMPV is capable of entering cells both at the plasma membrane and via endocytosis.^29–31^ Thus, the only partial inhibition of HMPV by statins (Figure 4) may be due to preserved viral entry at the cell surface that is not inhibited by endocytosis inhibitors. Statins are competitive inhibitors of HMG-CoA reductase, a rate-limiting step in the mevalonate pathway. In addition to their role in inhibiting cholesterol biosynthesis, statins also impact other cellular processes. Statins can inhibit prenylation of proteins as well as inhibit cholesterol biosynthesis. While there are no data on prenylation of HMPV proteins, prenylation of viral proteins is proven to be important for hepatitis D virus and thought to be important for HIV and HSV.^32^ Future studies may elucidate the mechanism of HMPV inhibition by statins.

Aurintricarboxylic acid, a topoisomerase inhibitor, has in vitro antiviral activity against a number of RNA viruses, mediated by inhibition of the viral RNA polymerase.^21–24^ Interestingly, the assay had a number of hits from the topoisomerase inhibitor class. These did exhibit some antiviral effect but also substantial cytotoxicity, suggesting that some of the antiviral effect occurs through antiproliferative mechanisms. However, the topoisomerase I inhibitor camptothecin exhibits antiviral activity against the positive-sense RNA virus enterovirus 71, mediated by topoisomerase I relocalization to the cytoplasm and interaction with the viral 3CD protein.^33^ Further studies are needed to explore the biologic relevance of topoisomerase inhibitor effects on HMPV.

These results show that the MTS is capable of identifying lead compounds with in vitro activity against HMPV. This method provides proof of concept for the capacity to screen much larger libraries. Further experiments will be needed to explore modifications of these drugs using medicinal chemistry to enhance their antiviral potency and identify the mechanisms of action. These studies offer promise to develop new antivirals against HMPV.

## Supplemental Material

Supplemental material for A medium-throughput screen for inhibitors of human metapneumovirus Click here for additional data file. Supplemental Material for A medium-throughput screen for inhibitors of human metapneumovirus by Jennifer C Becker, Sharon J Tollefson, David Weaver and John V Williams in Antiviral Chemistry and Chemotherapy
