# Supplementary material for: A medium-throughput screen for inhibitors of human metapneumovirus
Source: Antivir Chem Chemother. 2019 Feb 13;27:2040206619830197. doi: 10.1177/2040206619830197 (PMC6376503; doi:10.1177/2040206619830197)
Supplement: Supplemental material for A medium-throughput screen for inhibitors of human metapneumovirus [file Supplemental_Material.pdf]

Supplemental Table 1. Potential hits identified in screen.

PubChem compound name	Pubchem CID	Formula	Activity/Use
4-(Dimethylamino)-n-[7-(hydroxyamino)-7-oxoheptyl]benzamide	3994	C16H25N3O3	Histone deacetylase inhibitor
5-cis Iloprost	54313	C22H32O4	Prostacyclin analogue, eicosanoid, anti-inflammatory
5-Nonyloxytryptamine	1797	C19H30N2O	Serotonergic agonist
7-[4-(Dimethylamino)phenyl]-N-hydroxy-4,6-dimethyl-7-oxohepta-2,4-dienamide	5562	C17H22N2O3	Histone deacetylase inhibitor
Acacetin	5280442	C16H12O5	Bioflavonoid
Actinomycin D	457193	C62H86N12O16	RNA transcription inhibitor, anti-neoplastic
Amiodarone	349989353	C25H29I2NO3	Adrenergic agonist
Aurintricarboxylic acid	2259	C22H14O9	Dye, protein synthesis inhibitor
Aurothioglucose	6104	C6H11AuO5S	Antirheumatic
Benzyl-dimethyl-tridecylazanium	2330	C22H40N	Benzalkonium, antimicrobial
Busulfan	2478	C6H14O6S2	Alkylating agent, antineoplastic
Caudoside	120676	C30H44O9	Cardiac glycoside
Cetrimonium Chloride	8154	C19H42N	Quaternary ammonium, antimicrobial
Chlorhexidine Base	2713	C22H30Cl2N10	Cationic antimicrobial
Chlorprothixene	667466	C18H18ClNS	Thioxanthene, antipsychotic
Ciclopirox	2749	C12H17NO2	Protein synthesis inhibitor, antifungal
Cycloheximide	6197	C15H23NO4	Protein synthesis inhibitor
Digoxin	30322	C41H64O14	Cardiac glycoside
Dihydrocelastrol	10411574	C29H40O4	Pentacyclic triterpenoid
Emetine	10219	C29H40N2O4	Alkaloid, emetic, inhibits protein synthesis
Etoposide	36462	C29H32O13	DNA topoisomerase inhibitor, anti-neoplastic
Flecainide acetate	41022	C17H20F6N2O3	Sodium channel blocker, antiarrhythmic
Genkgetin	5271805	C32H22O10	Bioflavonoid
Gossypol	3503	C30H30O8	Polyphenolic aldehyde, male contraceptive
Hexachlorophene	3598	C13H6Cl6O2	Bisphenol topical antiseptic
Irinotecan hydrochloride	74990	C33H38N4O6	DNA topoisomerase inhibitor, anti-neoplastic
Isoproterenol Hydrochloride	5807	C11H17NO3	Beta adrenergic agonist
Itavastatin	5282452	C50H46CaF2N2O8	HMG-CoA reductase inhibitor
LPMXVESGRSUGHW-XPXORLBYSA-N	12420	C29H44O12	Cardiac glycoside
Magestin	4048	C24H32O4	Progestogen, antineoplastic
Mefloquine Hydrochloride	65329	C17H16F6N2O	Phospholipid-interacting antimalarial
Methotrexate	126941	C20H22N8O5	Antifolate, immunosuppressant
Methylbenzethonium	4135	C28H44NO2	Alkylbenzene, antimicrobial
Methylene blue cation	4139	C16H18N3S	Antimethemoglobinemic
Mevastatin	64715	C23H34O5	HMG-CoA reductase inhibitor
Mitomycin D	25028	C15H18N4O5	Antineoplastic
Mitoxantrone	4212	C22H28N4O6	Anthracenedione antibiotic, antineoplastic
N-Methyl-4-(3,4-dichlorophenyl)tetralin-1-amine	5203	C17H17Cl2N	5HT uptake inhibitor, antidepressant
Paclitaxel	36314	C41[14]C6H51NO14	Microtubule inhibitor, antineoplastic
Perhexiline	4746	C19H35N	Mitochondrial enzyme modulator, coronary vasodilator
Pimozide	16362	C28H29F2N3O	Dopamine antagonist, antipsychotic
Piroxicam	54676228	C15H13N3O4S	Non-steroidal anti-inflammatory
Quinacrine	237	C23H30ClN3O	Acridine antimalarial
Simvastatin	54454	C25H38O5	HMG-CoA reductase inhibitor
Teniposide	5396	C32H32O13S	DNA topoisomerase inhibitor, anti-neoplastic
Thioridazine	5452	C21H26N2S2	Phenothiazine, antipsychotic
Thiostrepton	16129638	C72H85N19O18S5	Cyclic peptide, antibacterial
Topotecan	60700	C23H23N3O5	DNA topoisomerase inhibitor, anti-neoplastic
Triclosan	5564	C12H7Cl3O2	Polychloro phenoxy phenol, antimicrobial
Unidigin	3061	C41H64O13	Cardiac glycoside
Valinomycin	5649	C54H90N6O18	Cyclododecadepsipeptide ionophore antibiotic
